# Determination of Tranquilizers in Swine Urine by Ultra-High-Performance Liquid Chromatography-Tandem Mass Spectrometry

**DOI:** 10.3390/molecules23123215

**Published:** 2018-12-05

**Authors:** Yingyu Wang, Xiaowei Li, Yuebin Ke, Chengfei Wang, Yuan Zhang, Dongyang Ye, Xue Hu, Lan Zhou, Xi Xia

**Affiliations:** 1 Beijing Advanced Innovation Center for Food Nutrition and Human Health, College of Veterinary Medicine, China Agricultural University, Beijing 100193, China; wangyingyu_1992@163.com (Y.W.); xiaowei@cau.edu.cn (X.L.); m18811587355@163.com (C.W.); zhangycau@163.com (Y.Z.); wintersunwyp@163.com (D.Y.); hututuhuhu@163.com (X.H.); m18482100424@163.com (L.Z.); 2 Shenzhen Center for Disease Control and Prevention, Shenzhen 518055, China; keyke@szu.edu.cn

**Keywords:** tranquilizers, UHPLC, MS/MS, swine urine

## Abstract

A rapid, reliable, and sensitive method was developed for the determination of ten tranquilizers in swine urine. Sample preparation was based on solid-phase extraction, which combined isolation of the compounds and sample cleanup in a single step. Separation was performed on a reversed phase C_18_ column by gradient elution with a chromatographic run time of seven minutes, consisting of 0.1% formic acid in water and acetonitrile as the mobile phase. Multiple reaction monitoring in positive mode was applied for data acquisition. Matrix-matched calibration was used for quantification and good linearity was obtained with coefficients of determination higher than 0.99. The average recoveries of fortified samples at concentrations between 0.05 and 10 µg/L ranged from 85% to 106% with interday relative standard deviations of less than 13% in all cases. The limits of detection and limits of quantification obtained for tranquilizers in the urine were in the ranges of 0.03–0.1 µg/L and 0.05–0.25 µg/L, respectively. The applicability of the proposed method was demonstrated by analyzing real samples; diazepam was detected at concentrations between 0.3 and 0.6 μg/L.

## 1. Introduction

Tranquilizers are frequently used to prevent mortality and loss of meat quality during transport from the farm to the slaughterhouse. They are also adopted to enhance the feed conversation ratio by reducing animal activity. However, their use has been regulated by setting maximum residue limits (MRL) or even through total prohibition in food-producing animals owing to the potential risk to consumers [1]. In China, residues of chlorpromazine, diazepam, and xylazine are not allowed in food of animal origin [2]. Similarly, the European Union Commission has already included chlorpromazine in the list of prohibited substances (Commission Regulation No 37/2010) [3].

Therefore, there is a need for rapid multiresidue analytical methods that cover a wide range of analytes to ensure compliance with legislation. For screening purposes, enzyme-linked immunosorbent assay [4] and chromatographic techniques such as high-performance liquid chromatography with ultraviolet detection (HPLC-UV) [5,6] have been used. For confirmatory purposes at trace levels, mass spectrometry (MS) is the generally accepted technique capable of offering characterized structures of analyte fragment ions. Thus, gas chromatography-mass spectrometry (GC-MS) methods with or without derivatization have been reported for the determination of tranquilizers in matrices of animal origin [7,8]. Moreover, because of its high selectivity and sensitivity, the combination of liquid chromatography and tandem mass spectrometry (MS/MS) has been frequently adopted to detect these drugs in animal tissues or plasma [9,10,11,12,13,14,15,16,17,18]. Based on ultra-high-performance liquid chromatography (UHPLC) and time-of-flight mass spectrometry (TOF MS), a multiclass method was developed for screening and quantification of veterinary drugs, including eight tranquilizers in milk [19]. However, very few methods have been published on the analysis of tranquilizer residues in urine samples. Olmos-Carmona and Hernández-Carrasquilla [8] described a GC-MS method for the determination of these drugs in urine, but sample preparation was relatively time-consuming, and the detection limits, ranging from 5 to 50 µg/L, were not satisfactory. Chèze et al. [20] developed an LC-MS/MS method for the forensic analysis of bromazepam, clonazepam, and metabolites in urine and hair, but the analytes covered by this method are limited. Chiuminatto et al. [21] reported a UHPLC-MS/MS method combined with online solid phase extraction (SPE) for determination of 42 therapeutic drugs and drugs of abuse in human urine. Diazepam, estazolam, and nitrazepam were included in this method with limit of quantitation between 2.14 and 5.00 µg/L.

In this study, we developed a rapid and sensitive method using UHPLC-MS/MS for the analysis of ten tranquilizer residues in swine urine. Sample preparation and instrument conditions were optimized to achieve high throughput determination and the method performance was validated. The applicability of the method was demonstrated by the analysis of real swine urine samples for the presence of the target compounds.

## 2. Results and Discussion

### 2.1. Optimization of UHPLC-MS/MS

Experiments to select the optimum MS conditions and appropriate ions were performed by infusing standard solutions at 500 μg/L. Both electrospray ionization (ESI) and atmospheric pressure chemical ionization probes were tested, and ESI in positive mode was adopted as the ionization technique owing to its sensitivity and easy handling. Full scan spectra were acquired to select precursor ions and optimize the cone voltage. In all cases, the protonated molecule (M + H)^+^ of each analyte was found to be the most abundant ion. Collision-induced dissociation mass spectra were then recorded for each analyte at various collision energies to select two major transition reactions for both identification and quantification purposes. Product ion spectra of each of the analytes are shown in Figure S1. For azaperone, the fragment at *m/z* 165 represents the elimination of the pyridinyl-piperazinyl part of the molecule. A subsequent elimination of -C_3_H_6_ from this fragment leads to the ion at *m/z* 123. A similar fragmentation pathway is observed for haloperidol (*m/z* 165 and *m/z* 123). For droperidol, the ion at *m/z* 194 results from tetrahydropyridine ring opening followed by the losses of -HC=CC_2_H_4_- and the benzimidazole ring. The ion at *m/z* 165 is obtained from the successive loss of -NHCH_2_. For chlorpromazine, the ion at *m/z* 246 results from the elimination of side chain -C_2_H_4_N(CH_3_)_2_, whereas *m/z* 86 originates from the loss of the phenothiazine ring. For perphenazine, the fragments of interest (*m/z* 171 and *m/z* 143) result from the loss of the phenothiazine ring followed by that of -C_2_H_4_. For xylazine, the ion at *m/z* 164 can be explained by splitting off the fragment -NC_3_H_6_- from the protonated molecule, whereas the fragment NHC_3_H_6_S- gives rise to the ion at *m/z* 90. For oxazepam, the consecutive losses of -OH and -CO afford the fragment ions at *m/z* 269 and *m/z* 241. For diazepam, the losses of Cl atom and -COCH_2_N- result in the ion at *m/z* 193, whereas *m/z* 154 originates from the losses of the benzene ring and -COCH_2_N-. For nitrazepam, the elimination of the nitro group accounts for the ion at *m/z* 236. A second loss of -COCH_2_N- gives the ion at *m/z* 180. For estazolam, the ion at *m/z* 267 originates from ring opening followed by the loss of -CH=N-. Subsequent losses of Cl atoms and -C=N- result in the fragment ion at *m/z* 205.

Chromatographic conditions were optimized to obtain a minimal run time. Different mobile phases consisting of methanol or acetonitrile as organic phase and water with different concentrations of formic or acetic acid (from 0.02% to 0.2%) were studied. Better sensitivity and shorter retention time were achieved when acetonitrile was used as an organic solvent in the mobile phase. Meanwhile, the addition of formic acid provided better results than acetic acid and it was used to improve the ionization efficiency. Different gradient profiles and column temperature and flow rate were investigated to obtain a rapid and reliable separation. With the optimized conditions, the chromatographic run time was 7 min, including cleaning and re-equilibration, which allowed for high throughput analysis of samples.

### 2.2. Sample Preparation

**Figure 1:**
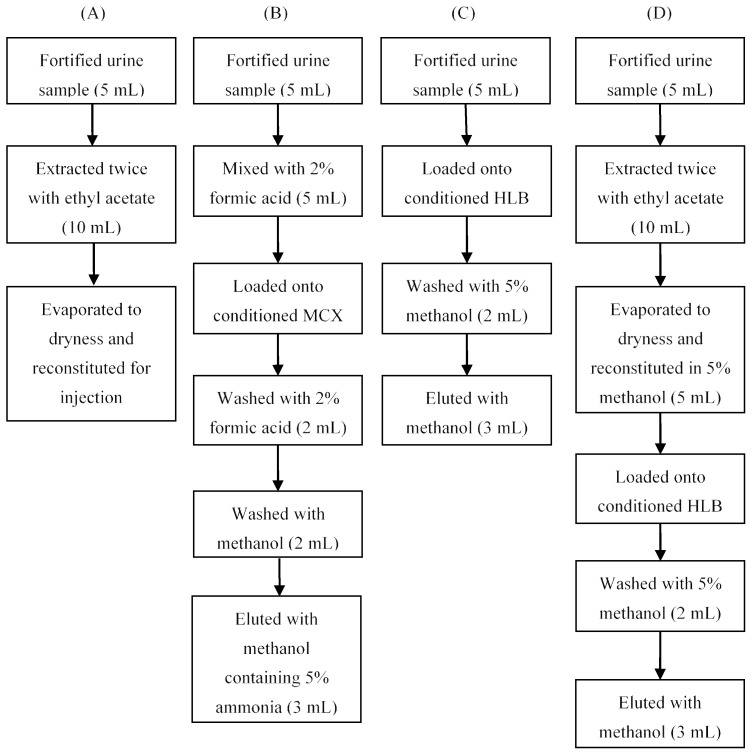
Optimization of sample preparation. HLB, MCX.

**Figure 2:**
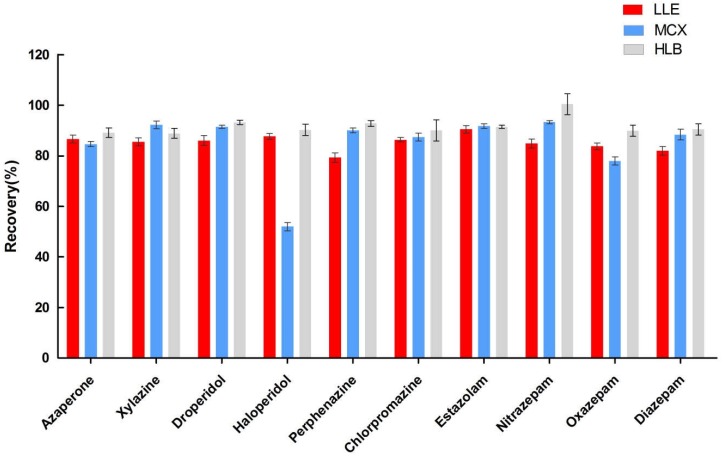
Recoveries of liquid-liquid extraction (LLE) and solid-phase extraction (SPE).

In the preliminary experiments, the effects of SPE and liquid-liquid extraction (LLE) were investigated for sample extraction and purification. Two different SPE cartridges, Oasis HLB and Oasis MCX, were tested using the procedures recommended by the manufacturer. Details of the procedure are shown as flow diagrams in Figure 1A–C. When using the MCX cartridge, the recoveries of some analytes (e.g., haloperidol) were lower than 60%. The HLB cartridge and LLE with ethyl acetate both gave acceptable recovery (Figure 2), but the cleanup effect of LLE was not satisfactory for all of the analytes. We then further investigated the performance of the combination of the HLB cartridge and LLE with ethyl acetate (Figure 1D). The addition of LLE before the SPE procedure did not improve the recovery or purification significantly, and it was very time-consuming. Therefore, the steps described in Figure 1C were adopted for sample preparation. The treatment of urine samples usually requires a hydrolysis step to release conjugated target compounds, but the results from previous studies [8,20] indicated that hydrolysis is not necessary for the analysis of tranquilizers, and consequently, we did not evaluate hydrolysis in this study.

### 2.3. Matrix Effects

**Figure 3:**
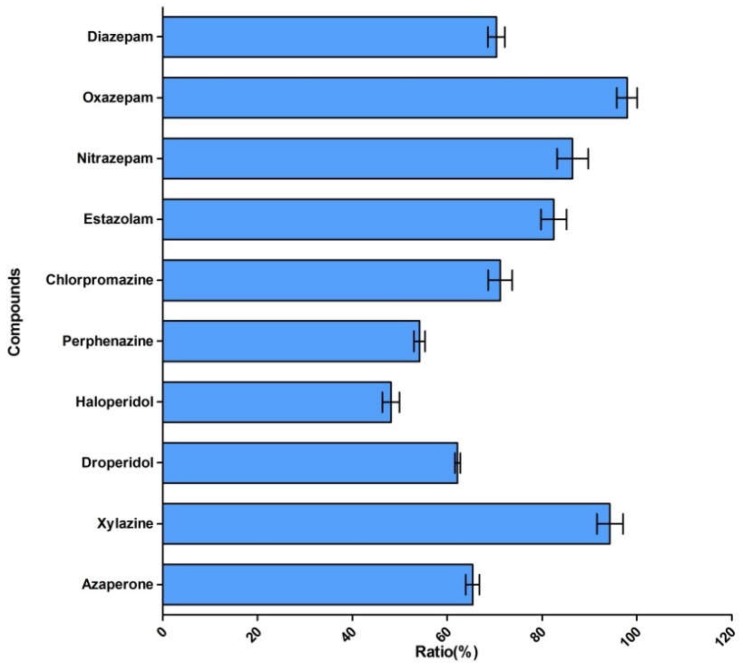
Ratio of matrix-matched standard versus standard in pure solvent.

Matrix-induced ion suppression was observed during method development. To evaluate the matrix effects, a matrix-matched standard and standard in pure solvent were prepared and analyzed. The ratios of matrix-matched standard versus standard in pure solvent for each analyte were as follows: haloperidol, perphenazine, 48–54%; azaperone, droperidol, chlorpromazine, diazepam, 62–71%; estazolam, nitrazepam, 82–86%; oxazepam, xylazine, 94–98% (Figure 3). To compensate for signal loss resulting from ion suppression, matrix-matched calibration curves were used for quantification.

### 2.4. Method Validation

**Figure 4:**
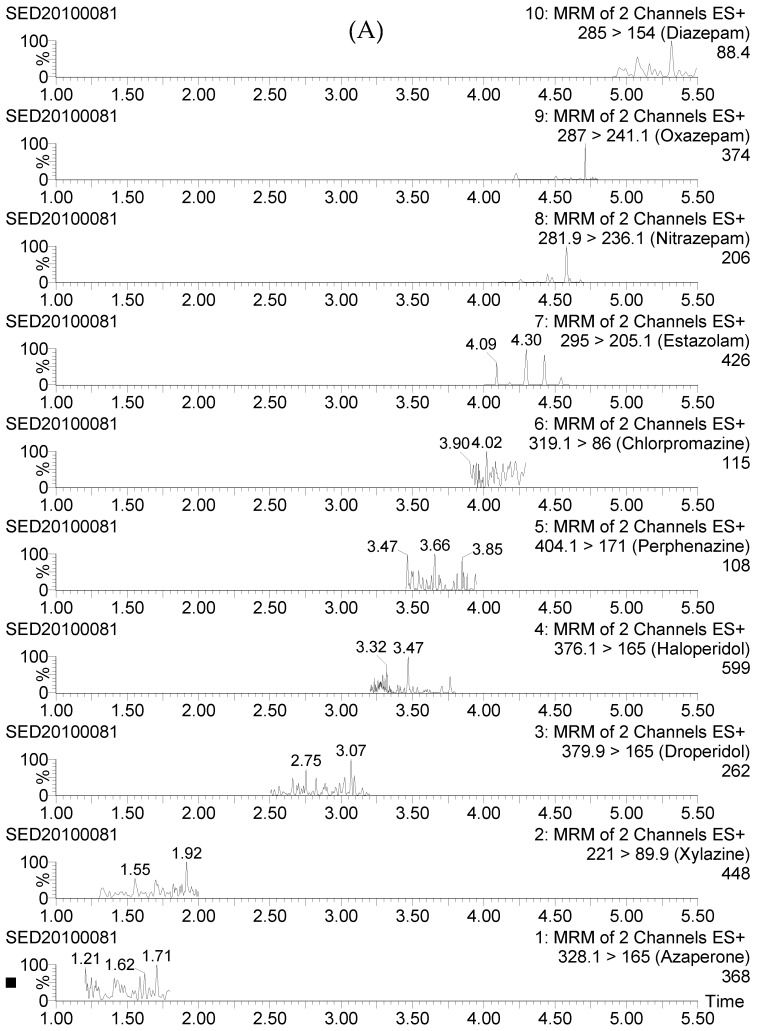

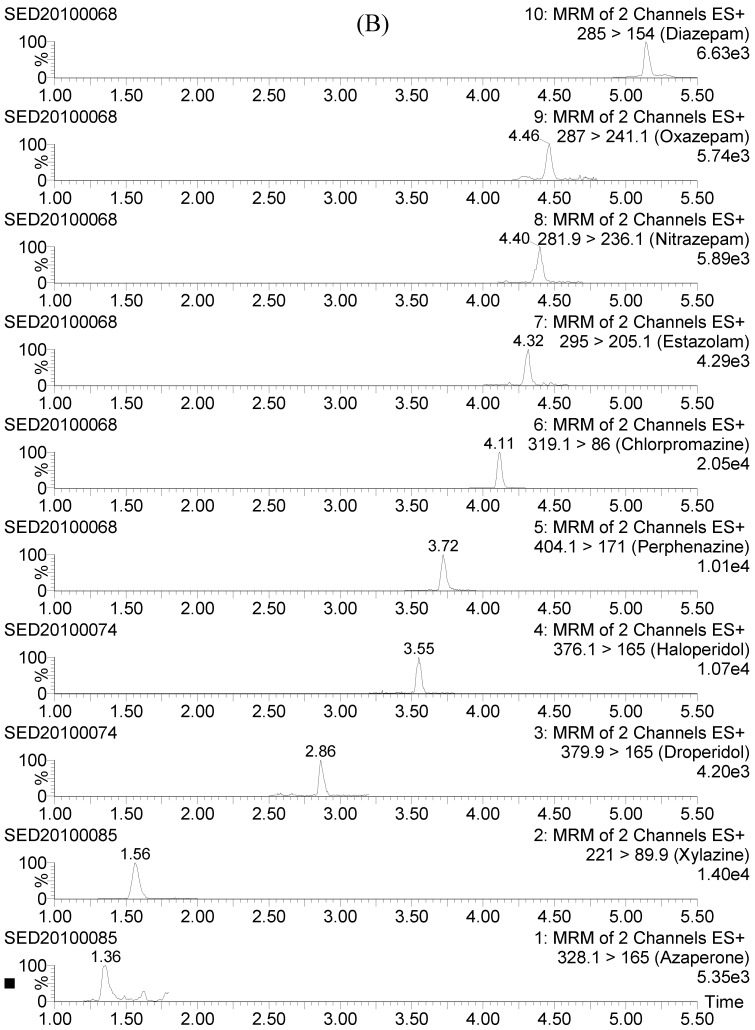

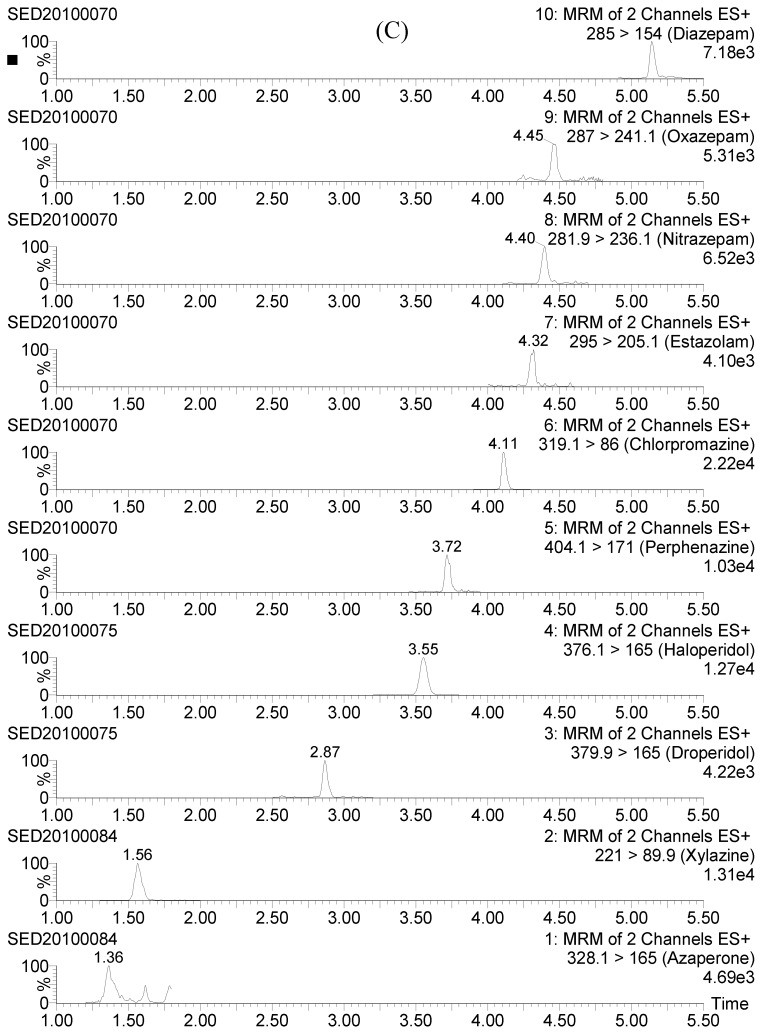
Typical ultra-high-performance liquid chromatography tandem mass spectrometry (UHPLC-MS/MS) chromatograms of blank sample (**A**), fortified sample (**B**), and matrix-matched standard (**C**).

Method specificity was demonstrated by the typical UPLC-MS/MS chromatograms of blank urine sample, as depicted in Figure 4A. An excellent linearity was observed for each analyte: Correlation coefficients (r^2^) were always above 0.992, while residuals were below 13% throughout the concentration range. Table 1 summarizes the mean recoveries and relative relative standard deviations (RSDs) of fortified urine samples. The recovery values of all analytes were in the range of 85–106% with interday RSDs of < 13%. Representative chromatograms of fortified urine sample and matrix-matched standard are presented in Figure 4B,C. The limit of detection (LOD) and LOQ of the method ranged from 0.03 to 0.1 μg/L and from 0.05 to 0.25 μg/L, respectively (Table 1).

### 2.5. Analysis of Real Samples

**Figure 5:**
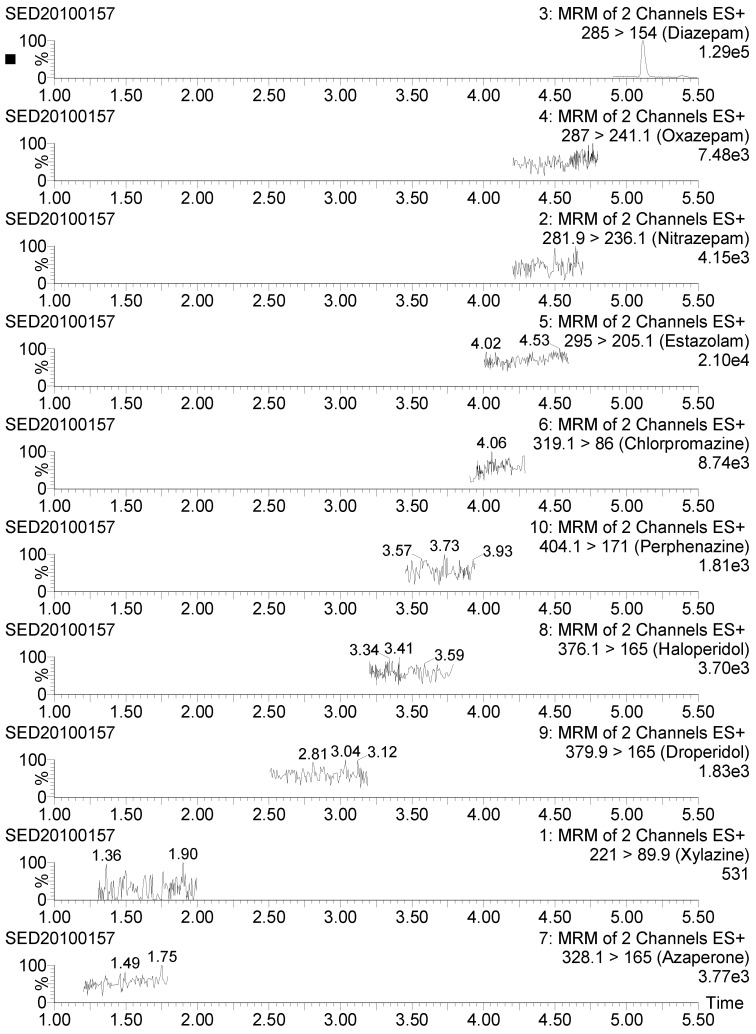
UHPLC-MS/MS chromatograms of a positive urine sample.

The applicability of the developed and validated method was evaluated by analyzing 30 urine samples from swine farms and five suspected swine urine samples from the Veterinary Drug Safety Inspection & Testing Center of Ministry of Agriculture (Beijing). The urine samples from swine farms were all free from tranquilizers, while diazepam was detected in three out of five suspected samples at concentrations of 0.3, 0.4, and 0.6 μg/L (Figure 5). The identification of the positive samples was confirmed by comparing the retention time and ion ratio of real samples to that of the matrix-matched standard.

## 3. Materials and Methods

### 3.1. Reagents and Materials

Azaperone, xylazine, and chlorpromazine were purchased from Sigma-Aldrich (St. Louis, MI, USA). Droperidol and haloperidol were obtained from Waco Corporation (Tokyo, Japan). Other standards (nitrazepam, estazolam, oxazepam, perphenazine, and diazepam) were provided by the National Institute for the Control of Pharmaceutical and Biological Products (Beijing, China). HPLC grade ethyl acetate, acetonitrile, and formic acid were purchased from Fisher Scientific Inc. (Pittsburgh, PA, USA). Oasis HLB (60 mg) solid phase extraction cartridges were supplied by Waters (Milford, MA, USA). Water was purified using a Milli-Q Synthesis system from Millipore (Bedford, MA, USA). A syringe filter (GHP ACRODISC, 0.2 µm) was purchased from Pall Corporation (Ann Arbor, MI, USA).

Individual stock standard solution was prepared in methanol at a concentration of 1000 mg/L. Mixed working standard solutions were prepared by subsequent dilution with methanol. These solutions were stored at −20 °C and were stable for at least six months.

### 3.2. Sample Preparation

Here, 5 mL of urine was transferred to a 50 mL polypropylene centrifuge tube. Fortification of samples for validation was carried out at this point by adding the mixed working standard solution and then equilibrating for 10 min. The sample was directly loaded onto the SPE cartridge previously conditioned with 2 mL of methanol and 2 mL of water. The cartridge was washed with 2 mL of 5% methanol in water and dried under vacuum for 2 min. The analytes were eluted with 3 mL of methanol and the eluate was evaporated to dryness under a gentle stream of nitrogen at 40 °C. The dried residue was reconstituted in 0.5 mL of 0.1% formic acid-acetonitrile (90:10, *v/v*) and filtered prior to UHPLC-MS/MS analysis.

### 3.3. UHPLC-MS/MS Analysis

Chromatographic separation was performed on a Waters Acquity ultra performance liquid chromatography system with column oven temperature maintained at 30 °C, using an Acquity BEH Shield RP18 column (50 × 2.1 mm I.D., 1.7 μm particle size; Milford, MA, USA). The mobile phase consisting of 0.1% formic acid in water (solvent A) and acetonitrile (solvent B) was pumped at a flow rate of 0.3 mL/min. The injection volume was 10 μL. The gradient elution program was as follows: 0–0.5 min, 90% A; 0.5–4.0 min, 90–60% A; 4.0–5.0 min, 60–0% A; 5.0–5.5 min, 0% A; 5.5–5.6 min, 0–90% A; 5.6–7.0 min, 90% A.

The UHPLC system was coupled to a Micromass Quattro Premier XE triple quadrupole mass spectrometer (Waters, Manchester, UK) fitted with an electrospray ionization (ESI) interface and controlled by MassLynx software (version 4.1, Milford, MA, USA). Typical ionization source parameters were: capillary voltage, 2.8 kV; source temperature, 100 °C; desolvation temperature, 350 °C; cone gas (N_2_) flow rate, 30 L/h, desolvation gas (N_2_) flow rate, 600 L/h. Collision-induced dissociation was performed using argon as collision gas at a pressure of 3.7 × 10^−3^ mbar in the collision cell. The multiple reaction monitoring (MRM) transitions and optimized cone voltages and collision energies are summarized in Table 2.

### 3.4. Method Validation

Twenty blank samples were prepared and analyzed to verify the absence of interfering substances around the retention time of analytes. The linearity of the method was evaluated by linear regression analysis of matrix-matched calibration curves at seven concentration levels (0, 1, 5, 10, 50, 100, and 500 μg/L) corresponding to 0–50 μg/L in urine samples. The recovery experiments were carried out to investigate the accuracy and precision of the method. Six replicates of spiked samples at three levels each were prepared on three different days. The precision, expressed as relative standard deviation (RSD), was determined by the intraday and interday assays. The LOD and LOQ were defined as the lowest concentrations with a signal-to-noise ratio (S/N) of 3 and 10, respectively.

## 4. Conclusions

We developed and validated a simple and sensitive method for the determination of tranquilizers residues in swine urine samples. The proposed method affords rapid analysis of tranquilizers accurately with minimal sample preparation. The separation and detection of ten tranquilizers is obtained using UHPLC with MS/MS detection within seven minutes, which makes this approach very amenable for high throughput regulatory monitoring of these compounds. The objective of the work was to develop a method for these residues in urine at sub μg/L levels; our results show that this has been successfully achieved.

## Figures and Tables

**Table 1:** Recovery, precision, and sensitivity of the method.

Compound	Fortified Level (μg/L)	Mean Recovery (%)	Intraday RSD (*n* = 6, %)	Interday RSD (*n* = 18, %)	LOD (μg/L)	LOQ (μg/L)
Azaperone	0.25	86.7	9.7	10.4	0.1	0.25
	1.0	89.5	3.2	12.1		
	10.0	91.2	6.8	8.5		
Xylazine	0.25	91.5	8.2	9.9	0.1	0.25
	1.0	86.7	3.9	8.8		
	10.0	88.3	9.4	10.5		
Droperidol	0.05	94.4	2.8	5.7	0.03	0.05
	1.0	92.7	5.8	9.7		
	10.0	92.6	8.5	10.1		
Haloperidol	0.05	89.2	5.3	8.0	0.03	0.05
	1.0	93.3	3.8	7.6		
	10.0	88.2	8.0	9.3		
Nitrazepam	0.1	93.8	5.0	7.2	0.05	0.1
	1.0	91.1	3.9	6.0		
	10.0	93.4	6.7	8.8		
Estazolam	0.1	95.7	10.1	10.9	0.05	0.1
	1.0	85.8	3.9	9.3		
	10.0	88.6	5.0	8.4		
Oxazepam	0.1	92.4	5.3	9.1	0.05	0.1
	1.0	91.0	4.5	11.0		
	10.0	90.9	6.7	10.8		
Perphenazine	0.1	106.2	7.5	12.9	0.05	0.1
	1.0	98.3	5.9	10.1		
	10.0	96.7	9.3	10.5		
Chlorpromazine	0.1	90.7	4.5	7.8	0.05	0.1
	1.0	92.1	7.3	10.3		
	10.0	86.9	9.1	10.9		
Diazepam	0.1	92.6	8.6	9.2	0.05	0.1
	1.0	91.4	4.3	8.1		
	10.0	87.3	8.1	9.0		

**Table 2:** MS/MS transitions and optimal conditions.

Compound	Precursor Ion (*m/z*)	Daughter Ions (*m/z*)	Cone Voltage (V)	Collision Energy (eV)
Azaperone	328	165*/123	30	21/35
Xylazine	221	90*/164	40	22/22
Droperidol	380	165*/194	30	27/16
Haloperidol	376	165*/123	40	25/37
Perphenazine	404	171*/143	40	22/28
Chlorpromazine	319	86*/246	30	19/23
Estazolam	295	205*/267	40	23/38
Nitrazepam	282	236*/180	40	24/37
Oxazepam	287	241*/269	40	24/15
Diazepam	285	154*/193	40	27/32

* Transitions for quantification.

## Supplementary Materials

The following are available online, Figure S1: MS/MS spectra of tranquilizers.
