# Supplementary figures and images for: Determination of Tranquilizers in Swine Urine by Ultra-High-Performance Liquid Chromatography-Tandem Mass Spectrometry
Source: Molecules. 2018 Dec 5;23(12):3215. doi: 10.3390/molecules23123215 (PMC6321033; doi:10.3390/molecules23123215)

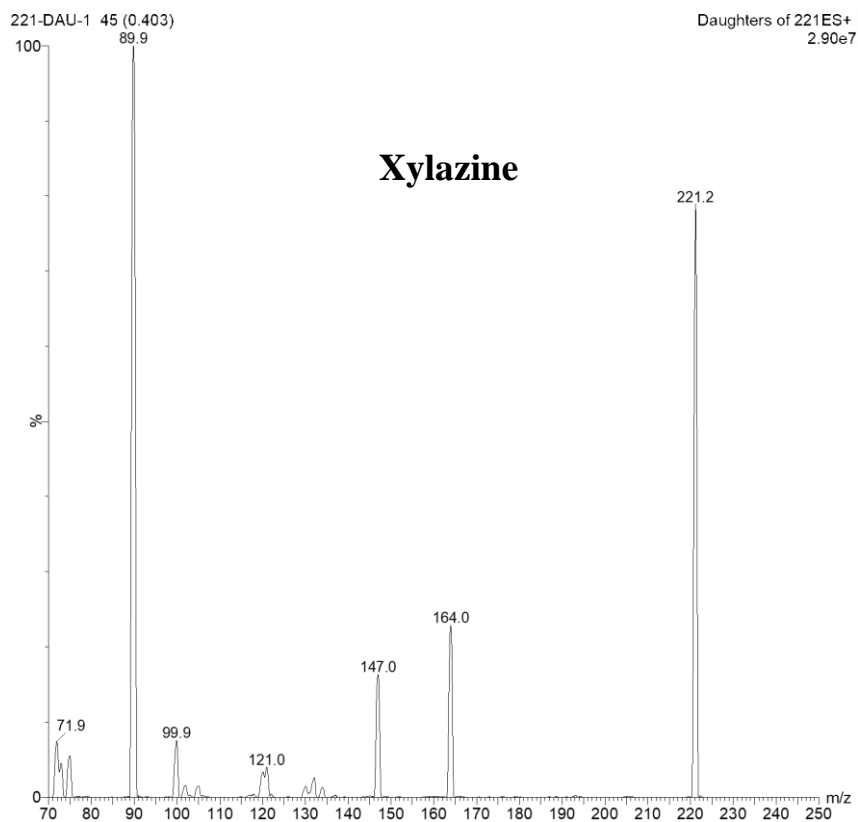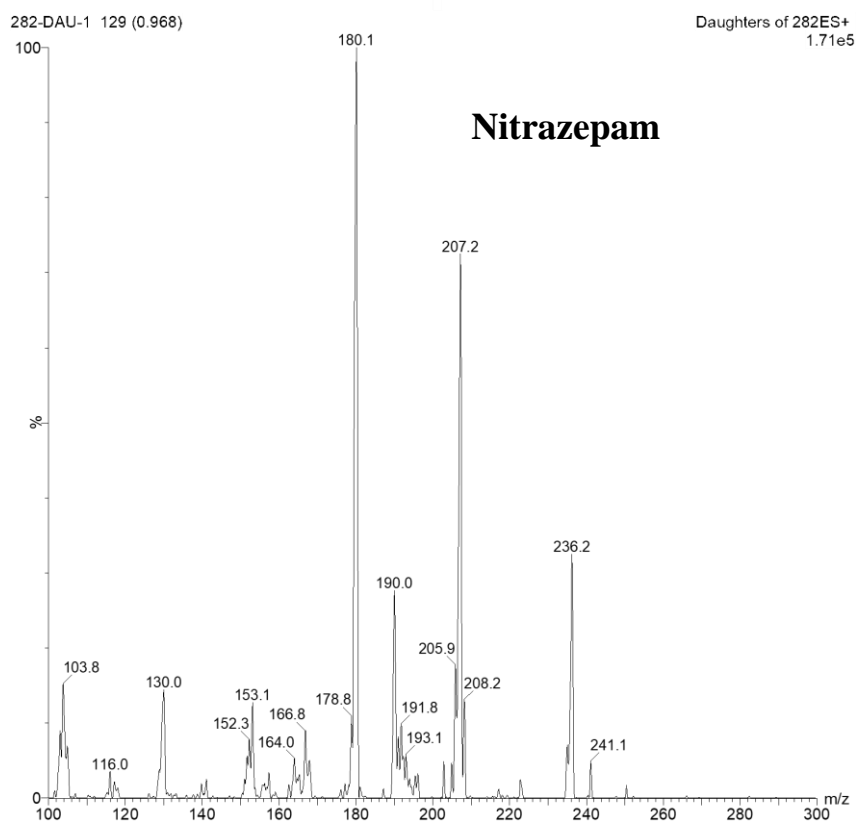

Figure S1. MS/MS spectra of tranquilizers.

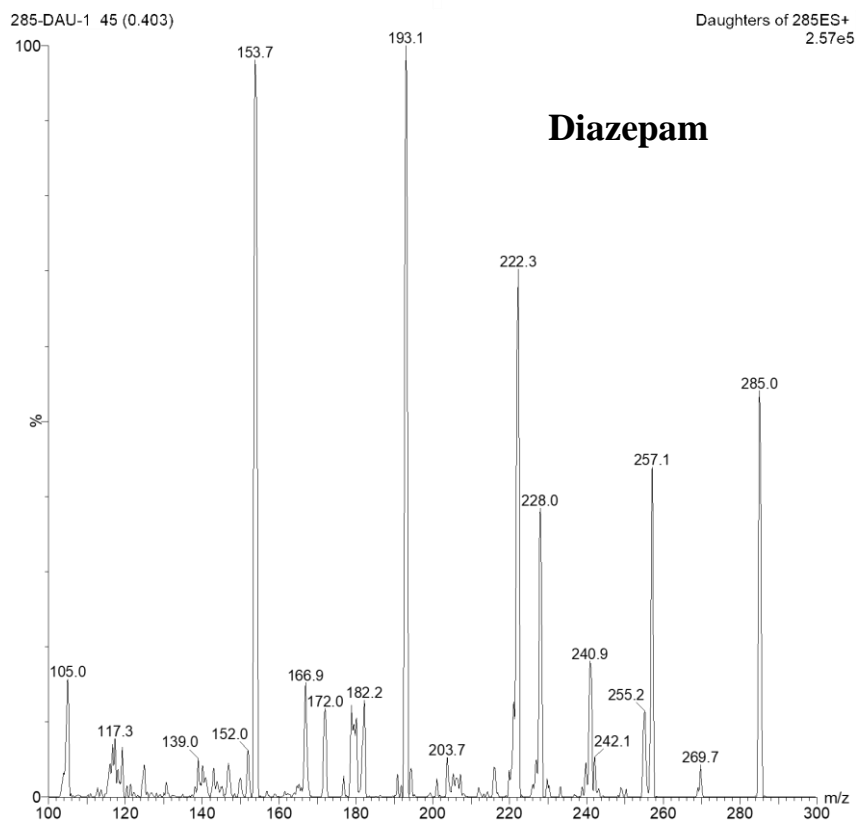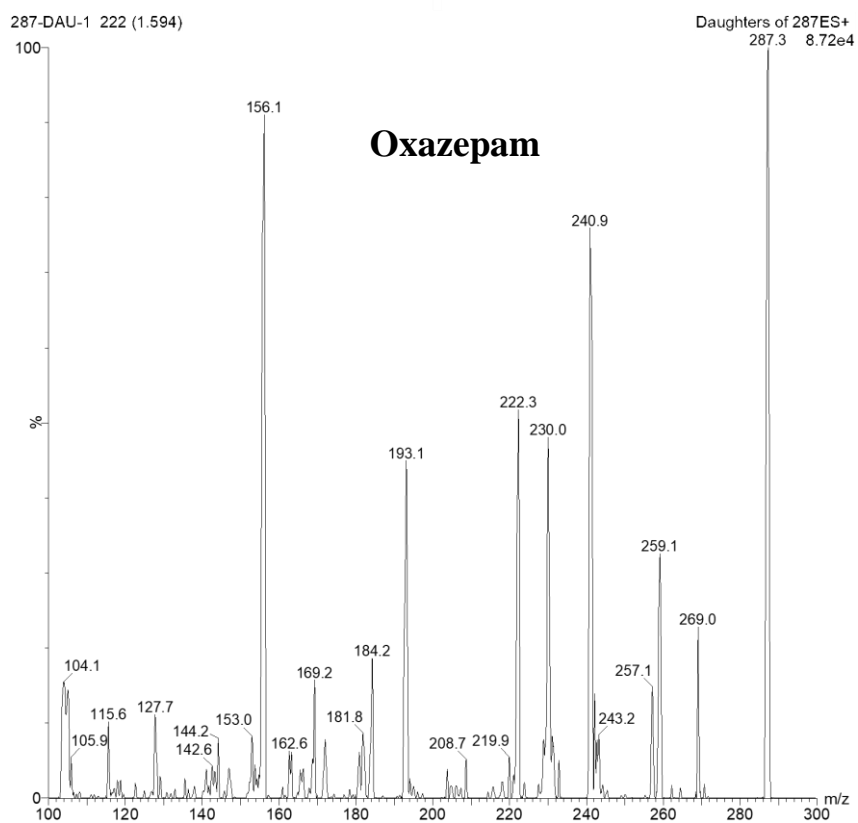

Figure S1. Cont.

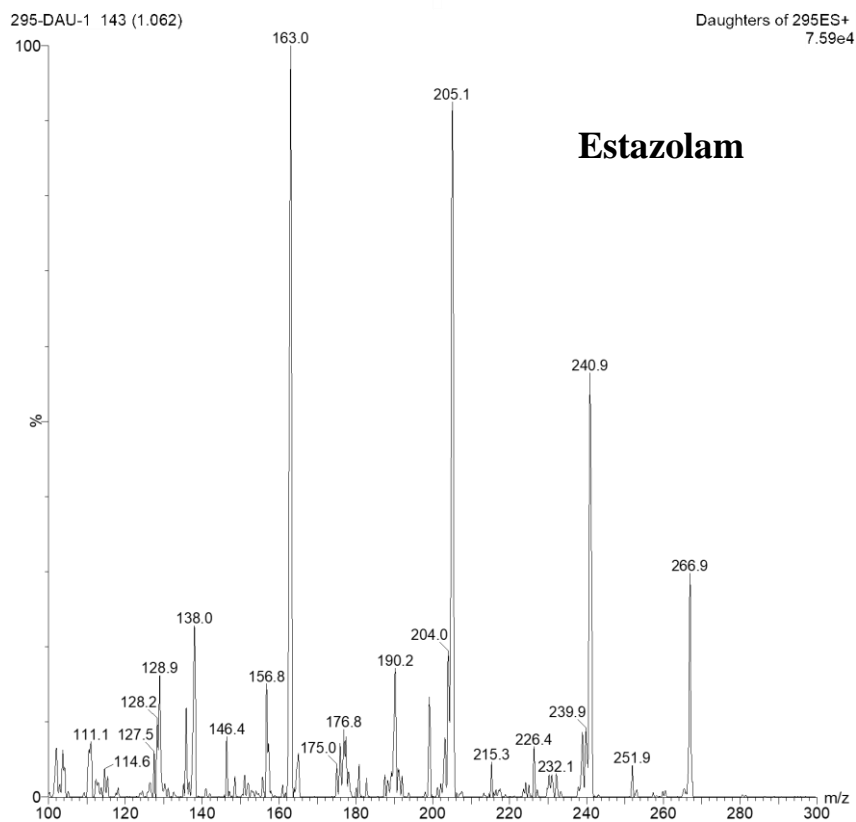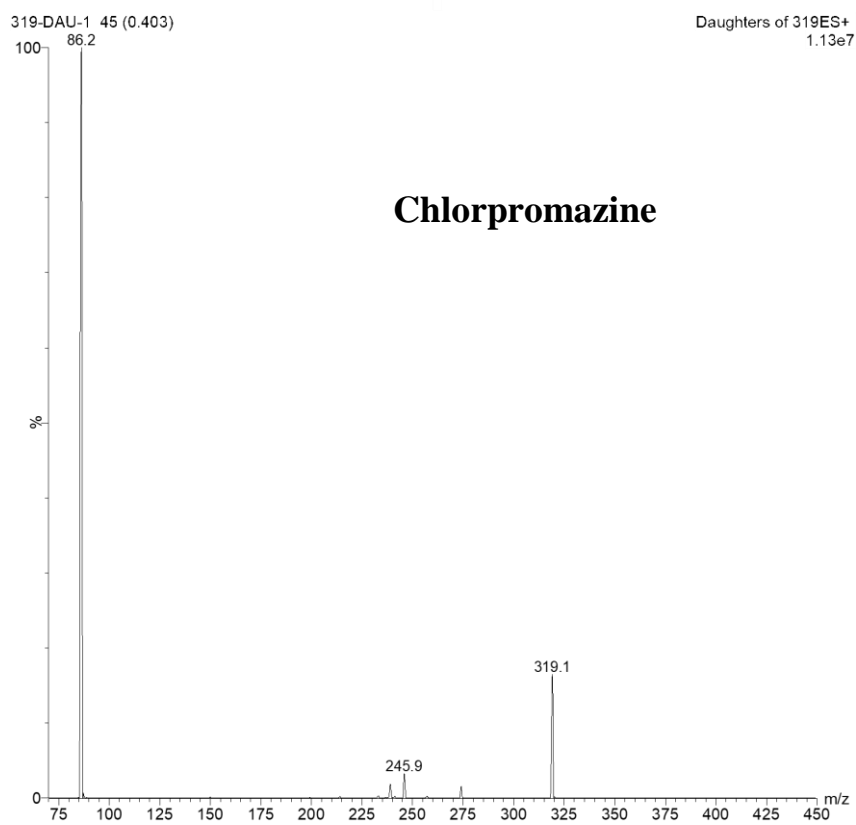

Figure S1. Cont.

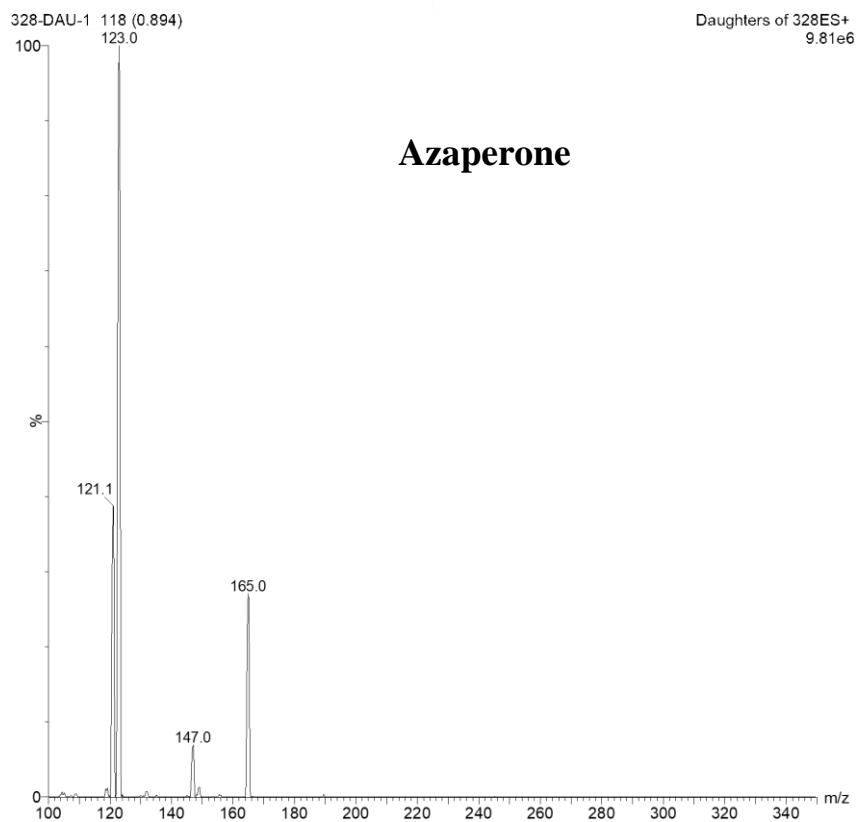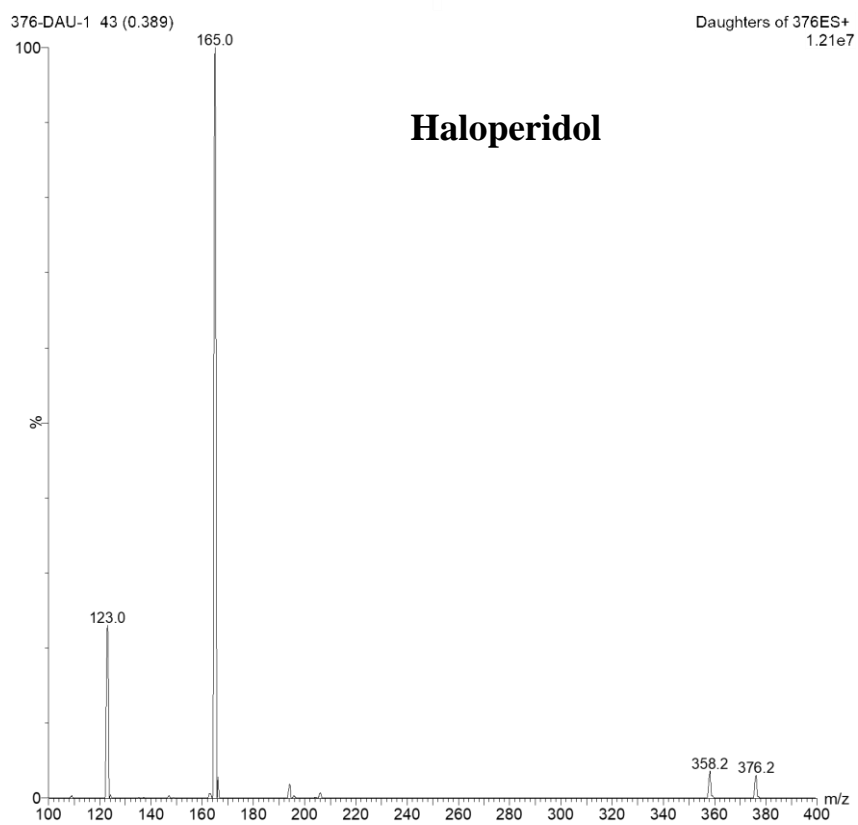

Figure S1. Cont.

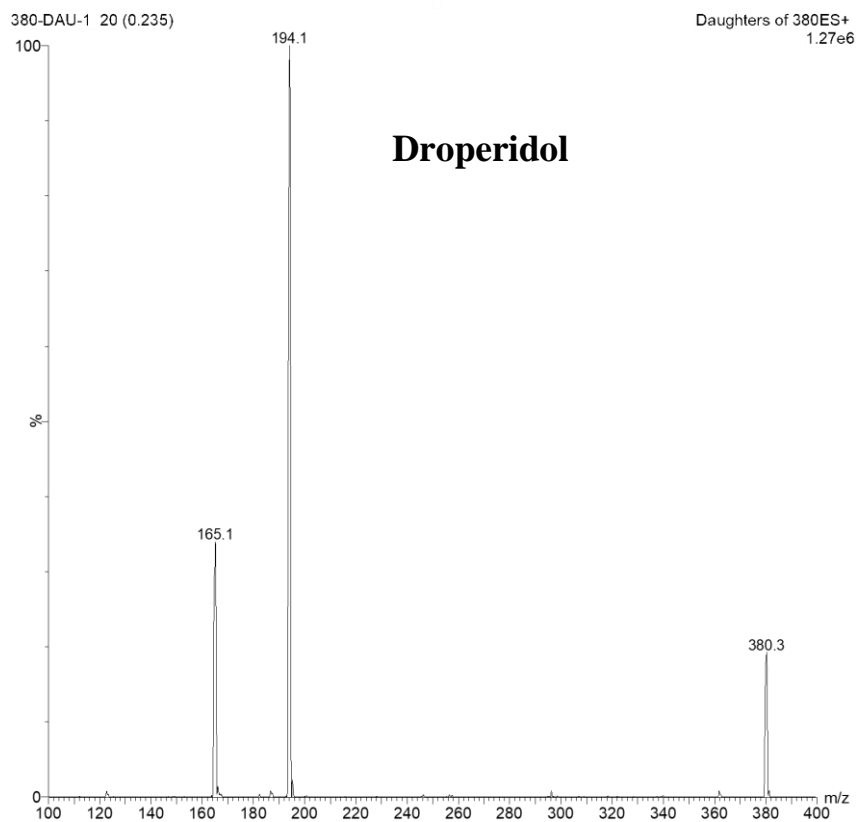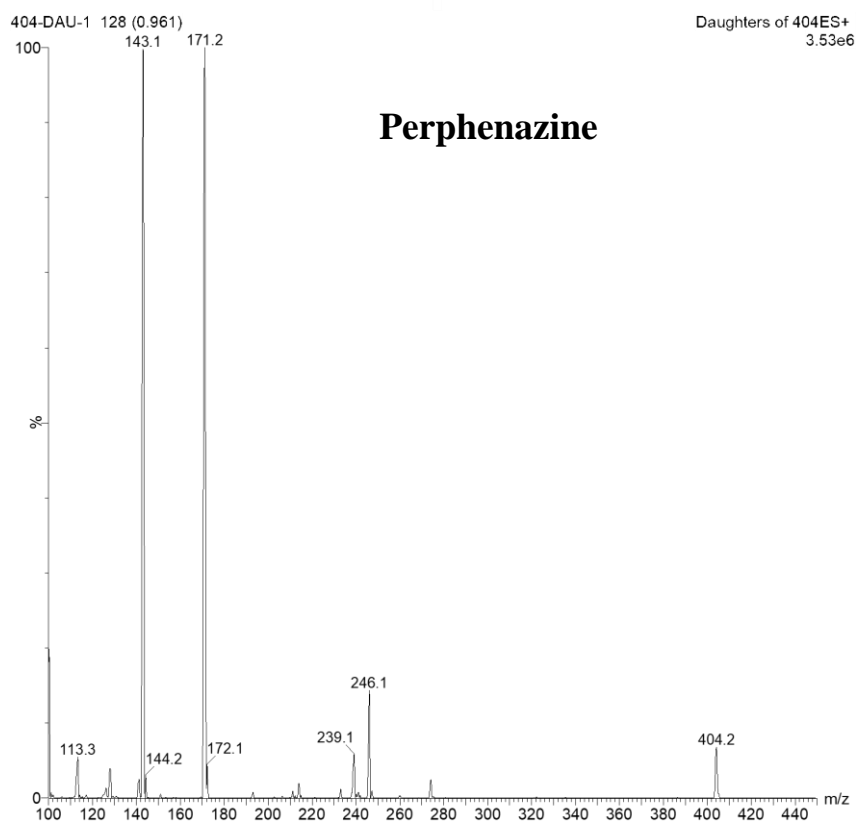

Figure S1. Cont.

Supplement: Supplementary file 1 [file molecules-23-03215-s001.pdf]
